# Supplementary material for: Predicting visual function by interpreting a neuronal wiring diagram
Source: Nature. 2024 Oct 2;634(8032):113–23. doi: 10.1038/s41586-024-07953-5 (PMC11446822; doi:10.1038/s41586-024-07953-5)
Supplement: Supplementary file 6 — CRF and ERF predictions for individual TmY4 and TmY9 cells. Analogous to Supplementary Data 3, but for TmY target types. Shown are the top four monosynaptic pathways, the strongest pathway passing through each of the top ten intermediary types (ranking from Extended Data Fig. 7), and the trisynaptic pathway Tm1–TmY–Dm3–TmY (see the section entitled Prediction of spatial normalization). [file 41586_2024_7953_MOESM6_ESM.zip › DataS4/TmY9qΓèÑ/720575940645162263.pdf]

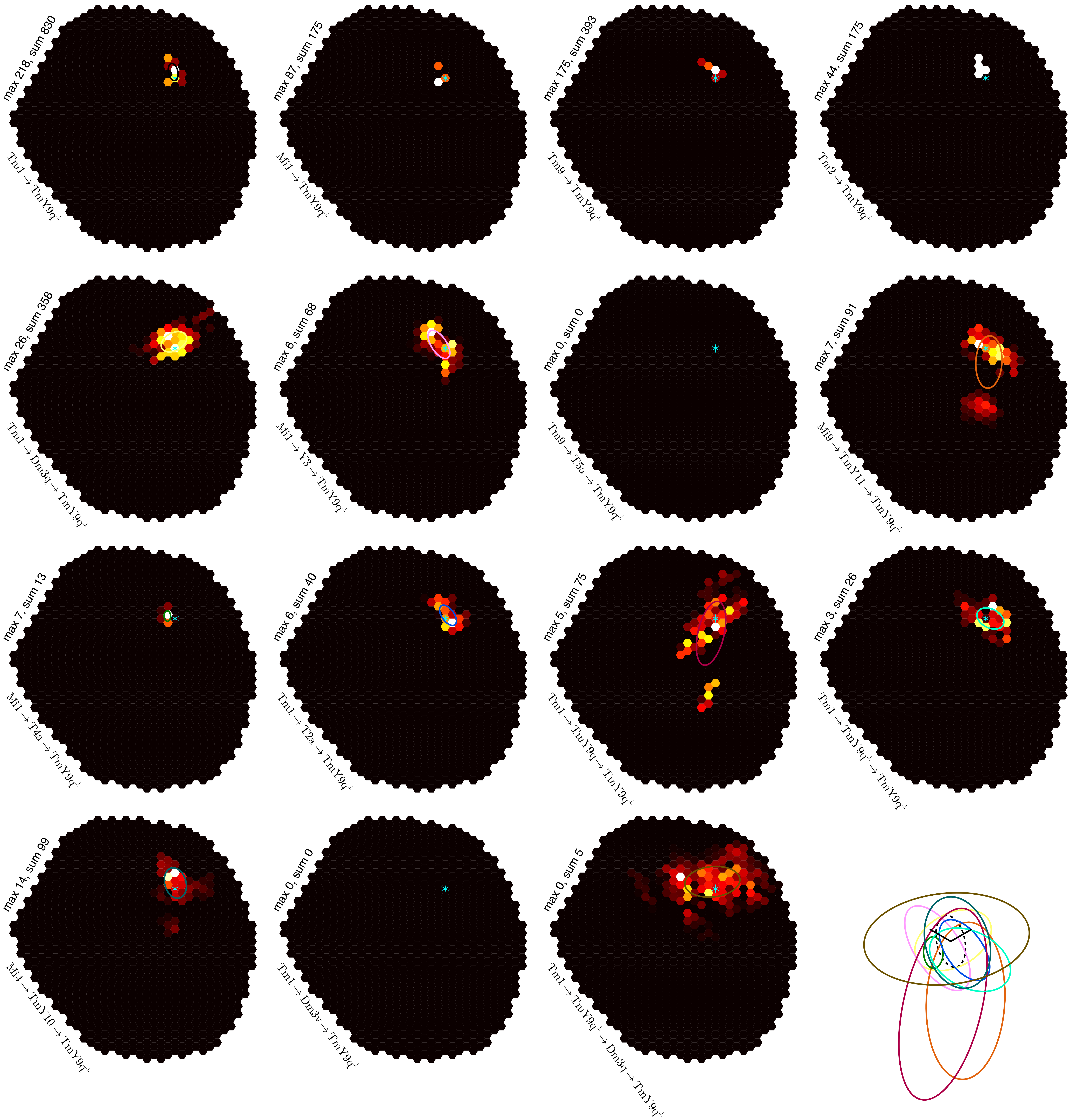

max 218, sum 830
Tm1 → TmY9q⊥
max 87, sum 175
Mi1 → TmY9q⊥
max 175, sum 393
Tm9 → TmY9q⊥
max 44, sum 175
Tm2 → TmY9q⊥
max 26, sum 358
Tm1 → Dm3q → TmY9q⊥
max 6, sum 68
Mi1 → Y3 → TmY9q⊥
max 0, sum 0
Tm9 → T5a → TmY9q⊥
max 7, sum 91
Mi9 → TmY11 → TmY9q⊥
max 7, sum 13
Mi1 → T4a → TmY9q⊥
max 6, sum 40
Tm1 → T2a → TmY9q⊥
max 5, sum 75
Tm1 → TmY9q → TmY9q⊥
max 3, sum 26
Tm1 → TmY9q⊥ → TmY9q⊥
max 14, sum 99
Mi4 → TmY10 → TmY9q⊥
max 0, sum 0
Tm1 → Dm3v → TmY9q⊥
max 0, sum 5
Tm1 → TmY9q⊥ → Dm3q → TmY9q⊥
